# The Frequency and Impact of Self-Imposed Elimination Diets on the Nutritional Status and Clinical Course of Disease in Children with Inflammatory Bowel Disease

**DOI:** 10.3390/nu15224712

**Published:** 2023-11-07

**Authors:** Ana Mocic Pavic, Sara Sila, Zrinjka Misak, Sanja Kolaček, Iva Hojsak

**Affiliations:** 1 Referral Center for Pediatric Gastroenterology and Nutrition, Children’s Hospital Zagreb, 10000 Zagreb, Croatia; sara.sila0810@gmail.com (S.S.); zrinjka.misak@gmail.com (Z.M.); sanja.kolacek@gmail.com (S.K.); ivahojsak@gmail.com (I.H.); 2 School of Medicine, University of Zagreb, 10000 Zagreb, Croatia; 3 Department of Pediatrics, University J.J. Strossmayer School of Medicine, 31000 Osijek, Croatia

**Keywords:** inflammatory bowel disease, children, elimination diet

## Abstract

Background and aims: From the patients’ perspective, diet has a relevant role in triggering symptoms of inflammatory bowel disease (IBD). There is a lack of prospective studies regarding the diet of children with IBD. The aim of this study was to assess the frequency and impact of self-imposed elimination diets on the nutritional status and clinical course of disease in the pediatric population. Methods: This was a prospective case-control study that included newly diagnosed patients with IBD and healthy controls (age/sex-matched peers and siblings) over a one-year period. The participants were examined in three categories: (1) anthropometric data and nutritional status; (2) dietary intake, as obtained by a Food Frequency Questionnaire (FFQ); and (3) dietary beliefs and elimination diets, as obtained by a structured questionnaire. Results: Overall, one-hundred and thirty-seven participants were included (twenty-eight with Crohn’s disease, sixteen with ulcerative colitis, three with IBD-unclassified, and seventy healthy controls). Only 15% of patients followed the self-imposed elimination diet upon the diagnosis, which increased to 47.6% by the end of the follow-up. The elimination diet did not influence the nutritional status and quality of the diet. Self-imposed elimination diets were not a risk factor for disease relapse. Most of the patients received nutritional counseling during the follow-up. Conclusions: The number of patients following self-imposed elimination diets had increased during the disease course but with no influence on nutritional status or relapse risk.

## 1. Introduction

The etiopathogenesis of inflammatory bowel diseases (IBDs) is unknown; however, available evidence suggests an interaction of genetic predisposition, the immune system, intestinal microbiota, the intestinal epithelial barrier, and the environment [1,2]. Diet is likely to play an important role in environmental risk factors, not only related to pathogenesis but also having an impact on the course of the disease and symptom severity. Patients often avoid different types of food on their own initiative, with the hope of improving their health or reducing the symptoms of the disease. However, the actual impact of diet on the onset of the disease and its course has not yet been determined. Previous studies, mostly performed in the adult population, have shown that 72 to 85% of adults with IBD adhere to some elimination diet [3,4,5,6,7]. Most often, they eliminate red meat, spicy foods, and alcohol but also unleavened vegetables, dairy products, seeds, and dietary fiber. The number of pediatric studies is very limited [8,9]. These studies have shown that around 36 to 50% of patients follow restrictive diets, mainly excluding gluten and dairy or decreasing carbohydrate intake.

However, none of these studies reported whether self-elicit elimination diets resulted in better symptom control and, more importantly, whether children with IBD were at nutritional risk and whether nutritional elimination contributed to altered nutritional status.

Considering the limited evidence on the self-imposed elimination diets of children with IBD, this study aimed to assess the frequency and impact of these diets on the nutritional status and clinical course of disease in children with IBD.

## 2. Methods

This was a prospective case-control study that included newly diagnosed patients with IBD being followed for a period of one year and healthy controls. Healthy controls were randomly selected from the elementary and secondary schools and were matched by age, gender, and area of residence with the IBD patients. The healthy controls had no chronic disease or gastrointestinal symptoms and did not follow any dietary restrictions. Written consent was obtained from the patients who were 9 years of age or older and one of their parents/guardians. In patients who were younger than 9 years of age, written consent was obtained only from their parents or caregivers.

As for the healthy controls, permission was obtained from the appropriate authorities; parents were informed about the survey by the school principals and teachers and their written consent was obtained. This study was approved by the Ethics Committee of Children’s Hospital Zagreb and the Central Ethical Committee of the University of Zagreb School of Medicine (No. 02-26/10-4-15, date 10 April 2015).

The IBD was diagnosed following the revised Porto criteria [10]. The severity of the disease has been estimated by the pediatric Crohn’s disease activity index (PCDAI) and pediatric ulcerative colitis activity index (PUCAI) [11,12].

The primary aim of this study was to estimate the frequency of self-imposed elimination diets in children with IBD and their impact on the nutritional status and disease course. A self-imposed elimination diet is any type of elimination diet initiated by the patients or their parents/guardians that was not advised by a health care professional. However, as a part of standard care, patients were followed by a pediatric gastroenterologist and dietician to ensure that they were not at nutritional risk. Reports on the proportion of patients who received detailed nutritional advice were collected. The secondary aims were to compare the difference in dietary intake and nutritional status between children with IBD and the healthy controls and to assess whether self-imposed elimination diets were risk factors for relapse. Relapse was defined for CD as a PCDAI score of >10 and for UC as a PUCAI score of >10; for all diseases, the need for the introduction of remission induction therapy was the definition. Anthropometric data and nutritional status were assessed by body height (BH), body weight (BW), body mass index (BMI), upper arm circumference (MUAC), and skinfold—above the triceps and subscapular muscle (TSF and SSF). An electronic scale was used to measure BW and a portable stadiometer was used for BH. The TSF and SSF were estimated using a Holtain skinfold caliper. Bioelectrical impedance was used to assess the subjects’ body composition (Maltron BF906, Maltron International Ltd., Rayleigh, Essex, UK) and the amount of fat and muscle tissue. Nutritional status was defined according to the criteria of the World Health Organization (WHO) [13]. The obtained results were compared with the standardized curves of the WHO for children and adolescents (5–19 years), where a Z-score for a BMI less than −2 standard deviations (SDs) indicates undernutrition, less than −3 SDs means undernourishment, above +1 SD means overweight, and above +2 SDs means obesity.

Food consumption data were obtained using a Food Frequency Questionnaire (FFQ) version which was previously validated for Croatian children and adolescents [14]. The FFQ that was used contained eighty-seven different food items divided into eight different food groups: “milk and milk products”, “cereals and grains”, “juices and sodas”, “fruits”, “vegetables”, “snacks”, ‘meat, poultry, eggs, and fat’, and ‘fast food’. The FFQ included frequently consumed national foods and estimated the frequency and quantity of the consumption of food items in the last month. Available frequencies of food consumption were “never”, “1–3 times a month”, “once a week”, “2–4 times per week”, “5–6 times per week”, “once a day”, “2–3 times per day”, “4–5 times per day”, or “6+ times per day”. Available portion sizes were small, medium, and large; simple portion sizes photos were used to distinguish the former. The frequency of consumption was obtained in the form of a personal interview with trained interviewers, with the presence of the caregivers (in children younger than 12 years of age) or without their presence in older participants. The intake of 24 nutrients was analyzed: total protein, plant protein, total fat, saturated fatty acids (SFAs), monounsaturated fatty acids (MUFAs), polyunsaturated fatty acids (PUFAs), cholesterol, total carbohydrates, mono- and disaccharides, polysaccharides, dietary fiber, sodium, potassium, calcium, magnesium, phosphorus, iron, zinc, retinol equivalent, vitamins B1 and B2, niacin, vitamin B6, and vitamin C. The results were compared with D-A-CH references [15].

A structured questionnaire was used to assess dietary beliefs and elimination diets. All patients underwent a structured interview/questionnaire conducted by the examiner consisting of 13 questions (Table 1). Questions related to eating habits and personal beliefs were compiled based on previously published studies on the adult population [5,6,16].

In IBD patients, all parameters were determined at recruitment, one year after the diagnosis, and at the onset of relapse during the follow-up period. In the control group, these parameters were determined on one occasion, at the inclusion into this study.

## 3. Statistical Analysis

Statistical analysis was performed with the IBM SPSS 26.0 program (Chicago, IL, USA). Qualitative and categorical variables are presented in terms of frequencies while quantitative variables are characterized by mean and standard deviation or median and range, depending on the distribution. The distribution of the data was analyzed using the Smirnov–Kolmogorov test and histograms. Differences in the categorical variables were determined by the χ^2^ test while, for differences in continuous variables, with respect to the distribution, parametric *t*-tests or nonparametric Mann–Whitney U tests were used for independent samples; for paired samples, either *t*-tests or nonparametric Wilcoxon tests were used. Correlations were determined, depending on the type of variables, by the Spearman or Pearson tests. Regression analysis was performed with the aim of assessing potential risk factors for relapse. First univariate binary logistic regression was used to determine factors for multivariate logistic regression. Values of *p* < 0.05 or a 95% confidence interval (CI) were considered significant.

## 4. Results

Epidemiological data. In this study, one-hundred and thirty-seven participants were included: forty-seven patients with IBD [(twenty-eight with Crohn’s disease (CD), sixteen with ulcerative colitis (UC), and three with IBD-unclassified (IBD-U)] and seventy healthy controls. Based on clinical indices, twenty-one (75%) patients with CD had a mild disease, four (14%) a moderate to severe disease, and three (11%) an inactive disease. In patients with UC, nine (56%) had a mild disease and seven (44%) had a moderate disease. The mean age of patients with IBD was 14.9 ± 0.4 years and did not differ significantly from the healthy controls (mean age 14.1 ± 0.2 years, *p* = 0.23). Overall, 19 (40.4%) in the IBD cohort were female vs. 38 (54%) among the healthy controls (*p* = 0.141).

**Figure 1:**
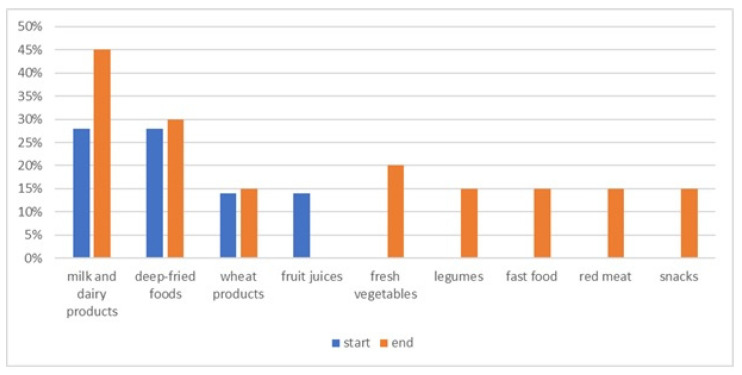
List of most often avoided foods at the beginning and the end of this study.

Primary outcome. Patient responses to the questionnaire are summarized in Table 1. At baseline, only five patients (15%) followed the elimination diet while, at the end of this study, that number increased to twenty (47.6%) (ten with CD, eight with UC, and two with IBD-U) (*p* = 0.027). The most common restricted foods at the beginning of this study were: milk and dairy products (28%), deep-fried foods (28%), white flour (14%), and fruit juices (14%). The number of restricted food groups significantly increased by the end of this study and they were milk and dairy products (45%), deep-fried foods (30%), fresh vegetables (20%), wheat products (15%), legumes (15%), fast food (15%), fresh juices (15%), red meat (15%), snacks (15%), etc. (Figure 1).

Table 2 shows the difference in dietary intake between groups with and without elimination diets. At the beginning of this study, the group with the elimination diet consumed more fruit while, for all other foods and nutrients, there was no significant difference. At the end of this study, a significant difference was found for the intake of unsaturated fatty acids, which was lower in the elimiation diet group, and the protein intake, which was higher in the elimination diet group.

At the beginning of this study, subscapular skin fold and lean mass were higher in children with the elimination diet (*p* = 0.021 and *p* = 0.048, respectively). At the end of this study, no significant difference was found in nutritional status.

The majority (n = 34, 72%) of all patients received detailed nutritional counseling during the follow-up, among which, 18/20 (90%) were on a self-imposed nutritional diet. Most stated that they received advice from a dietitian (20/34) and/or from a pediatric gastroenterologist (16/34). A total of sixteen (34%) patients also sought out nutrition information from other sources (internet, TV, patients’ association) and only four (8.5%) believe that information was provided exclusively through other sources (internet, TV, association) without professional advice. In addition, 20 patients (43%) received dietary supplements (enteral preparations—sip feeds or vitamins), of which, 15 were in the group with the elimination diet.

Secondary outcomes. At the diagnosis, IBD patients had significantly lower body weight and height Z-scores for age and sex compared to the healthy controls (Table 3). No difference in the BMI Z-scores for age and sex, TSF and SSF, as well as body composition assessed by bioelectrical impedance were seen (Table 3). At the diagnosis, IBD patients had lower energy intake, expressed as a percentage of the recommended daily intake corrected by age and sex (mean 90.1 ± 31.4 vs. 122.4 ± 46.9%; *p* < 0.001), compared to the healthy controls. A significantly lower intake at the diagnosis was seen for carbohydrates, calcium, and phosphorus in IBD patients compared to the healthy controls (Table 3).

After the year of follow-ups, the anthropometric data of children with IBD improved and there was no significant difference to the healthy controls anymore (body weight mean 0.1 ± 1.1 vs. 0.3 ± 1.2; *p* = 0.42; body height mean 0.3 ± 1.2 vs. 0.1 ± 1.1; *p* = 0.16; BMI Z-score mean −0.1 ± 1.1 vs. 0.3 ± 1.1; *p* = 0.23). Furthermore, no differences in the TSF and SSF, as well as the percentage of fat and muscle tissue, were seen as well (*p* > 0.05 for all parameters).

However, significant differences regarding dietary intake remained for the percentage of daily calories (*p* = 0.004), unsaturated fat (*p* < 0.0001), total carbohydrates (*p* = 0.029), calcium (*p* = 0.004), and phosphorus (*p* = 0.031), which were lower in IBD patients.

During their follow-up, 34% (n = 16) of children had a relapse of the disease. No potential risk factors for relapse—including age, sex, body mass and height BMI for age, disease activity index, CRP, albumin, energy intake, protein, fat, carbohydrate intake, and the use of an elimination diet—were identified as significant (Table 4).

## 5. Discussion

This is the first prospective case-control study on the incidence and the role of self-imposed elimination diets in children with IBD. It was revealed that a small proportion of patients started a self-imposed diet before the diagnosis, only 11%. However, this number increased four times during the first year after the diagnosis. Studies in adults showed that food elimination is common among patients with IBD who try to use them to alleviate symptoms but also to prolong the period of remission. In adult patients, the percentage of self-imposed elimination diets is high, up to 90% during the disease course [3,4,7,17]. As for the pediatric population, the first cross-sectional study was conducted back in 1998 by Green and co-workers [18]. The study included 154 children with IBD and found that even 90% of patients with CD and 71% with UC reported at least one diet change since the diagnosis. Another similar study from Poland confirmed that 61.5% of the 155 included children/parents avoided at least one group of foods for fear of worsening the disease [19]. Our study was the first one to have included newly diagnosed patients and assessed diet elimination, not only during the course of the disease but also before the diagnosis was made.

The types of foods that are mainly eliminated by children and adults with IBD are milk and dairy products, baked and fried foods, fresh vegetables, legumes, wheat products, snacks, and sweets [5,20,21]. Based on the data from this study, we found similar results; mainly milk and dairy products, fried/baked foods, and fresh vegetables were eliminated.

As patients with IBD are at higher risk for nutritional deficit due to the disease itself, the question is whether these self-imposed diets increase the risk even more. It is known that various dietary restrictions can result in unbalanced food intake with a deficiency of certain macro- and micronutrients, which can have negative short and long-term effects, especially on the growth and development of children. The results of this study showed that there was no statistical t difference in macro- and micronutrient intake between the group of patients who started with elimination and those who did not, except for there being a higher intake of unsaturated fatty acids and a lower intake of proteins at the end of this study in patients without elimination. To the best of our knowledge, none of the previous pediatric studies evaluated these parameters. In the adult population, a cross-sectional study from Korea [22], which included 104 patients with the disease, showed a reduction in the vitamin A, zinc, and fiber intake in the elimination diet group while, for other macro and micro nutrients, no difference was found. Previous studies in adults have shown that patients who used elimination diets were more frequently malnourished [22,23]. We were not able to confirm these results using the data from this study. This could be at least partially explained by the study design, which was, in our case, prospective; at the diagnosis, patients had a poorer nutritional status than the healthy controls and only a small proportion of patients were on the elimination diet. So, we can only speculate that altered nutritional status was mainly attributed to the disease itself. At the end of this study, however, the nutritional status of all patients improved and did not differ from the healthy controls. At that time, 43% of patients eliminated at least one group of foods from their diet but, still, this did not have any repercussions on their nutritional status. This is probably caused by the proper nutritional advice given to a great majority (90%) of patients who started elimination during the disease course. Adequate nutritional advice and guidance could correct any deficiencies in their diet by either a more balanced diet and/or food supplements. Indeed, though the majority of patients were on the elimination diet, even 75% of them were taking supplements.

Most patients on the elimination diet strive for a healthier diet; so, as many as 20% of respondents avoid fast food, sweets, and snacks. So far, no other studies have reported on this outcome.

Studies on adult patients strongly emphasize the patients’ distress regarding eating a normal diet, fearing that this could elicit the relapse/symptoms. A study by Limdi et al. showed that almost half of the patients were convinced that diet is a risk factor for developing the disease [3]. On the contrary, in a study conducted by Zallot et al., only 15.6% of patients believed that diet could trigger the disease [6]. This percentage was also small in our research; only 15% of patients at diagnosis believed that diet is the trigger of the disease, which was further reduced to only 8% during the disease course. On the contrary, more than 50% of our patients at the end of this study avoided certain foods to prevent relapse.

This was a prospective study; so, we were able to monitor whether some dietary modifications were associated with the relapse; however, none of the changes were shown to be risk factors for the disease relapse. In our cohort, neither the introduction of a self-initiated diet, modification of macronutrient intake, nor changes in nutritional status have been shown to precede relapse. To date, there are no studies in children; so, we cannot compare these results to other pediatric data. In one prospective adult study, meat, particularly red and processed meat; alcoholic beverages; and higher protein intake were associated with an increased likelihood of relapse in patients with UC [16]. A cross-sectional study of 103 adults with IBD showed a 79% lower risk of disease relapse in the highest quartiles of legume and potato intake compared to the lowest [24]. This discrepancy could be explained by the fact that the number of included patients in our cohort was much lower; however, our study was the only prospective study so patients were regularly monitored and controlled, assuring better insight into their diet at the time of relapse. Certainly, possible associations should be further explored in a large prospective, multicenter study.

It was reported previously that people with IBD most often seek nutrition information via the Internet or social media [25,26]. This was not the case in our study, where the percentage was much lower. Furthermore, an even smaller percentage, only 8.5%, believed that they received real information from these sources. This can be explained by the fact that in our center, the dietician is available to all patients with IBD and is involved in therapeutic care, especially in children with CD. As previously mentioned, supervision is probably the reason why children, despite having modified their diet, had no nutritional deficit.

Lastly, we should mention the possible shortcomings of this research. The first and probably the most important limitation is the relatively small number of patients and the even smaller number of children on an elimination diet at the time of diagnosis. However, this limitation is at least partially corrected by the fact that only newly diagnosed patients were included, this study was prospective, and the whole cohort was followed for one year. Second, the elimination diet questionnaire was not standardized. However, this questionnaire has straightforward unambiguous questions and it was used in previous research. Third, there is an issue of the potential impact of selective misreporting on the observed differences between the patients and the control group as the patients and their parents/caregivers were likely much more aware of their diet than the control group.

In conclusion, the number of patients that start with self-imposed elimination diets increased during the disease course; although, patients and their parents do not believe that diet has a major impact on their disease course. Furthermore, these dietary restrictions did not influence the quality of the diet or nutritional status which could be at least partially explained by the fact that the great majority of patients received professional nutritional counseling. Therefore, personalized nutritional counseling and ongoing nutritional assessment are warranted in all children with IBD.

## Figures and Tables

**Table 1:** Dietary beliefs and behavior of patients with inflammatory bowel disease at the beginning and the end of this study.

		Start n (%)	End of this Study (%)	*p*
Do you follow a special diet for your IBD?	1. yes 2. no 3. no response	5 (11) 40 (85) 2 (4)	20 (43) 22 (47) 5 (11)	0.027
Do you use food supplements?	1. yes 2. no 3. no response	4 (9) 40 (85) 3 (6)	29 (62) 14 (30) 4 (8)	0.048
Do you believe that nutrition is a trigger for disease?	1. yes 2. no 3. no response	7 (15) 40 (85)	4 (8) 36 (77) 7 (15)	0.002
Do you think that diet has a more important role than medicines in your disease course?	1. yes 2. no 3. no response	2 (4) 45 (96)	6 (13) 35 (74) 6 (13)	<0.001
Did you modify your diet since diagnosis?	1. yes 2. no 3. no response		28 (59) 14 (30) 5 (11)	
Do you avoid certain food groups to prevent relapse?	1. yes 2. no 3. no response		25 (53) 17 (36) 5 (11)	
Do you feel that the disease affects your appetite and food enjoyment?	1. yes 2. no 3. sometimes 4. no response		11 (23) 6 (13) 25 (53) 5 (11)	
During a relapse, do some food groups calm your symptoms?	1. yes 2. no 3. no response		8 (17) 34 (72) 5 (11)	
Do you share the same meal with your family?	1. yes 2. no 3. sometimes 4. no response		22 (47) 3 (6) 17 (36) 5 (11)	
Have you ever received nutrition advice?	1. yes 2. no 3. no response		34 (72) 8 (17) 5 (11)	
How would you indicate your appetite during relapse (on a scale of 1–10, mean ± SD)?			5.6 ± 1.3	
How would you indicate your appetite during remission (on a scale of 1–10, mean ± SD)?			9.4 ± 1	

**Table 2:** The difference in nutritional intake between groups with and without the self-imposed elimination diet at the beginning and the end of this study.

	START			END		
Median (Range)	Elimination Diet Group (n = 5)	Non-Elimination Diet Group (n = 40)	*p* Value	Elimination Diet Group (n = 20)	Non-Elimination Diet Group (n = 22)	*p* Value
Energy intake/kJ	7848.2 (4256.3–11,345.3)	7914.5 (3154.4–17,247.4)	0.471	7221.7 (4081.5–14,601.5)	8519.7 (3785.4–18,594.3)	0.208
Energy intake/kcal	1876.2 (1030.3–2712.0)	1904.0 (757.0–4146.8)	0.449	1801.7 (988.3–3489.8)	2062.3 (917.2–4596.0)	0.217
EER (%)/kcal	78.3 (51.5–104.3)	90.3 (37.9–165.9)	0.296	82.6 (44.2–134.2)	85.5 (45.9–199.7)	0.365
Total proteins (g)	87.7 (43.7–122.4)	80.4 (25.5–191.6)	0.847	87.8 (39.0–174.3)	87.3 (30.4–208.3)	0.562
Protein intake (% EI)	18.0 (15.8–20.5)	17.2 (13.5–26.1)	0.249	18.8 (15.4–22.5)	16.5 (13.3–20.8)	0.002
Vegetable proteins (g)	25.7 (12.9–32.0)	23.0 (10.2–62.8)	0.875	22.8 (7.0–37.8)	22.3 (11.0–64.9)	0.614
Animal proteins (g)	33.0 (21.1–72.5)	38.7 (7.2–106.5)	0.687	43.2 (14.0–118.1)	44.1 (12.3–114.7)	0.782
Total fat (g)	82.1 (41.6–104.1)	83.9 (21.1–192.7)	0.407	83.6 (29.9–148.3)	93.2 (36.9–278.5)	0.237
Fat intake (% EI)	36.4 (34.5–49.8)	41.1 (25.1–54.1)	0.875	40.13 (23.4–47.2)	39.4 (19.5–66.0)	0.743
Saturated fat (g)	20.8 (13.6–46.7)	28.8 (6.4–71.9)	0.331	27.6 (13.6–64.9)	39.6 (13.2–90.7)	0.062
Unsaturated fat (g)	25.4 (14.2–46.2)	38.0 (11.1–97.0)	0.080	13.3 (6.0–45.9)	28.6 (8.2–70.1)	0.003
Linoleic acid (g)	9.2 (6.1–842.9)	10.1 (3.6–780.3)	0.515	-	-	-
Cholesterol (g)	222.4 (132.0–331.0)	197.8 (66.5–888.5)	1.000	243.2 (35.0–879.0)	206.7 (49.8–746.1)	0.706
Total carbohydrates (g)	146.7 (120.9–249.2)	224.9 (99.9–476.0)	0.118	191.8 (96.6–401.1)	230.8 (108.0–507.6)	0.166
Carbohydrate intake (% EI)	47.0 (21.6–48.9)	47.3 (16.3–65.0)	0.349	46.5 (31.9–61.4)	47.9 (35.8–67.0)	0.940
Mono- and disaccharides (g)	67.9 (38.5–171.8)	86.3 (27.6–247.1)	0.562	76.6 (28.9–177.5)	95.6 (40.5–263.9)	0.632
Polysaccharides (g)	83.2 (25.7–180.2)	119.5 (32.6–237.7)	0.181	120.0 (51.0–268.3)	135.7 (70.9–312.4)	0.166
Fibre (g)	17.2 (0.0–20.9)	18.1 (0.0–43.5)	0.234	20.1 (7.0–33.0)	19.2 (11.0–64.2)	0.529
Sodium (mg)	1507.6 (881.3–2900.0)	1918.4 (519.3–5924.4)	0.314	1607.7 (760.0–4403.7)	2158.6 (635.0–4665.6)	0.247
Potassium (mg)	2526.6 (1488.1–6629.5)	2709.7 (1403.1–11,644.4)	0.958	2819.5 (1417.0–6629.5)	2601.0 (1502.8–6631.5)	0.435
Calcium (mg)	415.8 (330.0–1283.4)	690.9 (178.0–1591.2)	0.349	714.2 (142.0–1633.5)	644.2 (280.8–1418.0)	0.650
%DACH ref	34.7 (27.5–107.0)	62.8 (14.8–132.6)	0.314	59.5 (11.8–136.1)	53.7 (23.4–128.9)	0.706
Magnesium (mg)	211.9 (110.3–689.1)	197.9 (67.3–1318.9)	0.820	234.2 (98.4–689.1)	206.2 (77.9–640.3)	0.302
Phosphorus (mg)	1196.1 (698.8–2055.6)	1381.9 (407.3–2546.4)	0.449	1283.2 (510.0–2466.4)	1397.1 (630.0–2810.0)	0.546
%DACH ref	95.7 (55.9–164.5)	110.6 (32.6–203.7)	0.407	102.7 (40.8–197.3)	111.8 (50.4–224.8)	0.546
Iron (mg)	12.4 (6.0–16.4)	11.2 (4.1–28.2)	0820	11.5 (5–18.0)	11.6 (7.3–31.1)	0.687
%DACH ref	103.0 (40.1–136.5)	84.3 (27.2–234.8)	0.903	77.0 (41.7–149.7)	91.7 (48.4–259.2)	0.420
Zinc (mg)	6.7 (2.9–9.2)	6.7 (1.9–19.9)	0.539	7.1 (3.5–14.6)	7.4 (3.8–16.5)	0.669
%DACH ref	75.3 (40.9–103.5)	83.6 (26.4–215.3)	0.562	97.8 (42.1–147.6)	80.5 (37.5–174.7)	0.513
R.E. (ug)	1074.4 (880.9–1600.0)	892.5 (271.8–2232.8)	0.407	1080.8 (331.1–2074.8)	839.6 (129.2–2669.2)	0.497
Retinol (ug)	314.2 (0.1–646.2)	214.8 (0.0–1380.8)	0.903	147.1 (0.0–840.3)	220.0 (3.7–2172.8)	0.087
Carotenes (ug)	2647.5 (1383.8–4611.2)	2931.4 (286.4–8856.8)	0.793	4384.6 (1407.3–8948.7)	1903.3 (411.8–9855.4)	0.028
Vitamin B1 (mg)	3.7 (2.8–4.2)	3.6 (1.1–9.4)	0.958	3.7 (1.7–7.2)	3.6 (1.7–10.5)	0.614
Vitamin B2 (mg)	4.2 (2.7–5.1)	4.0 (1.4–9.5)	0.986	4.0 (1.4–7.4)	4.1 (1.6–10.6)	0.562
Niacin (mg)	35.8 (21.9–71.3)	35.8 (9.6–117.5)	0.636	39.7 (13.0–121.0)	31.5 (9.6–417.0)	0.801
Vitamin B6 (mg)	4.2 (2.9–4.6)	3.7 (1.2–9.9)	0.958	4.0 (1.5–7.5)	3.7 (1.5–10.8)	0.900
Vitamin C (mg)	171.4 (139.5–250.4)	155.3 (38.4–549.1)	0.661	164.0 (42.3–298.0)	132.6 (38.4–693.4)	0.420

**Table 3:** The difference in anthropometric data, body composition, and nutritional intake between patients with inflammatory bowel disease (IBD) at the diagnosis and the healthy controls.

	IBD (n = 47) Mean (SD)	Healthy Controls (n = 70) Mean (SD)	*p* Value
Body weight for age Z-score	−0.4 (1.3)	0.3 (1.2)	0.002
Body height for age Z-score	−0.4 (1.2)	0.1 (1.1)	<0.001
BMI for age Z-score	0.4 (1.1)	0.3 (1.1)	0.06
Mid-upper arm circumference for age Z-score	−0.5 (0.9)	−0.3 (0.8)	0.130
Skinfold above the triceps muscle for age Z-score	0.5 (0.8)	0.7 (0.8)	0.354
Skinfold above the subscapular muscle for age Z-score	0.4 (0.8)	0.6 (0.8)	0.138
Fat mass (%)	19.4 (7.7)	21.1 (8.5)	0.305
Lean mass (%)	79.9 (8.4)	78.9 (8.5)	0.529
Energy intake/kJ	8223.6 (2998.0)	10,306.2 (4301.8)	0.013
Energy intake/kcal	1986.6 (728.3)	2501.2 (1046.2)	0.011
EER (%)/kcal	90.1 (31.4)	122.4 (46.9)	<0.001
Total proteins (g)	86.7 (34.2)	105.7 (43.6)	0.029
Protein intake (% EI)	17.5 (2.4)	17.1 (2.8)	0.779
Vegetable proteins (g)	24.1 (10.4)	30.7 (16.1)	0.022
Animal proteins (g)	44.1 (21.9)	55.6 (28.6)	0.049
Total fat (g)	89.3 (36.5)	108.9 (56.9)	0.164
Fat intake (% EI)	40.1 (6.1)	38.4 (8.2)	0.184
Saturated fat (g)	32.3(14.9)	43.7 (23.0)	0.013
Unsaturated fat (g)	41.8 (18.1)	53.4 (28.5)	0.026
Linoleic acid (g)	46.4 (167.1)	11.8 (6.4)	0.182
Cholesterol (g)	250.6 (152.4)	303.1 (222.7)	0.319
Total carbohydrates (g)	225.1 (88.0)	303.4 (133.0)	0.001
Carbohydrate intake (% EI)	46.1 (8.8)	48.9 (9.2)	0.106
Mono- and disaccharides (g)	100.6 (56.2)	138.8 (64.1)	0.002
Polysaccharides (g)	122.7 (49.1)	154.2 (78.8)	0.037
Fibre (g)	19.3 (9.0)	25.5 (11.7)	0.009
Sodium (mg)	2095.4 (880.3)	2327.9 (1049.1)	0.305
Potassium (mg)	3239.7 (1843.6)	3990.1 (1984.3)	0.049
Calcium (mg)	702.2 (329.4)	1019.4 (492.2)	0.000
%DACH ref	62.2 (30.7)	86.3 (41.5)	0.002
Magnesium (mg)	241.2 (198.2)	293.7 (205.1)	0.253
Phosphorus (mg)	1354.0 (487.8)	1741.1 (705.5)	0.003
%DACH ref	111.3 (42.2)	139.3 (56.4)	0.015
Iron (mg)	12.3 (5.2)	14.8 (6.7)	0.060
%DACH ref	96.6 (43.9)	110.8 (53.2)	0.230
Zinc (mg)	7.6 (3.6)	8.9 (3.8)	0.155
%DACH ref	88.9 (40.5)	109.8 (47.5)	0.031
R.E. (ug)	1114.3 (541.2)	1380.8 (953.6)	0.156
Retinol (ug)	317.6 (303.9)	349.2 (577.8)	0.817
Carotenes (ug)	3206.4 (1823.3)	3947.4 (3167.6)	0.295
Vitamin B1 (mg)	4.1 (1.9)	4.8 (2.8)	0.250
Vitamin B2 (mg)	4.4 (1.9)	5.2 (2.8)	0.189
Niacin (mg)	50.3 (31.3)	59.8 (53.8)	0.375
Vitamin B6 (mg)	4.3 (1.9)	4.9 (2.8)	0.368
Vitamin C (mg)	186.5 (120.6)	243.2 (164.8)	0.087

**Table 4:** Potential risk factors for disease relapse (univariate analysis).

	HR	*p* Value	95% CI
Age (years)	0.596	0.716	0.75–1.22
Male sex	0.5	0.273	0.145–1.727
BM Z-score (SD)	1.155	0.572	0.7–1.91
BH Z-score (SD)	1.269	0.41	0.72–2.24
BMI Z-score (SD)	1.080	0.752	0.65–1.80
Triceps skinfold (SD)	1.293	0.534	0.58–2.91
Subscapular skinfold (SD)	1.327	0.513	0.57–3.1
Lean body mass (%)	1.022	0.566	0.95–1.10
Energy intake (kJ)	1.000	0.846	1.0–1.0
Protein intake (% energy intake)	1.000	0.911	0.99–1.01
Fat intake (% energy intake)	1.005	0.898	0.93–1.09
Carbohydrates intake (% energy intake)	0.987	0.711	0.92–1.06
Self-imposed elimination diet	1.333	0.768	0.19–8.99
Enteral supplementation	1.500	0.736	0.14–15.87

## Data Availability

Data are contained within the article.
